# Urinary microRNA in Diabetic Kidney Disease: A Literature Review

**DOI:** 10.3390/medicina59020354

**Published:** 2023-02-13

**Authors:** Chin-Chan Lee, Chia-Chun Chen, Cheng-Kai Hsu, Yih-Ting Chen, Chun-Yu Chen, Kai-Jie Yang, Ming-Jui Hung, I-Wen Wu

**Affiliations:** 1 Department of Nephrology, Chang Gung Memorial Hospital, Keelung 204, Taiwan; 2 Molecular Medicine Research Center, Chang Gung University, Taoyuan 333, Taiwan; 3 Department of Laboratory Medicine, Chang Gung Memorial Hospital at Linkou, Taoyuan 333, Taiwan; 4 Department of Cardiology, Chang Gung Memorial Hospital, Keelung 204, Taiwan; 5 College of Medicine, Chang Gung University, Taoyuan 333, Taiwan

**Keywords:** diabetes mellitus, diabetic kidney disease, exosomes, microRNA, urinary

## Abstract

Diabetic kidney disease is the most common primary disease of end-stage kidney disease globally; however, a sensitive and accurate biomarker to predict this disease remains awaited. microRNAs are endogenous single-stranded noncoding RNAs that have intervened in different post-transcriptional regulations of various cellular biological functions. Previous literatures have reported its potential role in the pathophysiology of diabetic kidney disease, including regulation of Transforming Growth Factor-β1-mediated fibrosis, extracellular matrix and cell adhesion proteins, cellular hypertrophy, growth factor, cytokine production, and redox system activation. Urinary microRNAs have emerged as a novel, non-invasive liquid biopsy for disease diagnosis. In this review, we describe the available experimental and clinical evidence of urinary microRNA in the context of diabetic kidney disease and discuss the future application of microRNA in routine practice.

Diabetes mellitus (DM) is a major health threat involving 463 million persons globally [1,2]. Diabetic kidney disease (DKD) constitutes the top cause of end-stage kidney disease worldwide [3,4]. The medical burden is complicated by its strong association with cardiovascular diseases, death [5,6], as well as elevated medical financial demand [7]. Improvements in the understanding of pathophysiology and early prediction of DKD remain an unmet clinical need.

Knowledge of systems biology and advances in high-throughput sequencing technology have revolutionized the understanding of the pathophysiology of DKD and biomarker development [8]. Clinical translation of different omic biomarkers for the prediction of DKD remains limited in terms of sample size and validation cohort. An approach that precisely identifies predisposed high-risk DM patients to renal progression is urgently awaited.

Optimal biomarkers have to provide adequate sensitivity and specificity, be obtainable in a non-invasive nature and assay with friendly laboratory skills using minimal time and economical consumption. Urine represents an ideal non-invasive biomarker, mainly for genitourinary diseases, because it is easily collected in large quantities without injury to the patient. In addition to varied types of proteins, the exosomes, which can be secreted by cells of different nephron segments, may also transport protein, mRNA, and microRNA (miRNA) markers produced in conditions of kidney malfunction or structural damage [9]. From them, the urinary exosomes can provide a panoramic view of the whole urinary system.

In this narrative literature review, we examine emerging evidence from experimental and human research that suggest the potential role of urinary miRNA for clinical application in the context of DKD.

## 1. Introduction of microRNA

The miRNAs are important epigenetic regulators of gene expression that intervene in various cellular processes of health and disease status. Genes encoding miRNAs are situated in the noncoding region or in introns of either protein-coding genes (miR-trons) or noncoding RNA [10]. These are small, less than 70 nucleotide stem–looped structures transcribed by RNA polymerase II located in the nucleus. miRNAs rarely bind to the coding regions of mRNA or genomic DNA, including promoter regions. However, they can actively play a role as regulators of cellular crosstalk by modifying their transcriptional program [11]. Currently, there are more than 38,000 mature sequences of miRNA included in miRbase, and the entry list is still growing [12].

Most miRNAs remain stable and have a prolonged half-life, but other individual miRNAs can experience a rapid decline in certain cellular circumstances because of the existence of specific environmental stimuli or cellular factors [13]. miRNAs are ubiquitous and can be present in different compartments of the body, including blood, urine and other body fluids. To avoid the degradation of miRNA by ribonucleases, they are often packaged in the form of micro-vesicles or exosomes or carried by RNA-binding proteins [14]. The miRNAs are potentially superior biomarkers than proteins and mRNAs because of their stability in body fluids and the high reproducibility by using accurate and sensitive amplification methods. However, the isolation and quantification of miRNAs are technique and time laborious, which limits their applications in routine clinical practice.

## 2. miRNAs in Kidney Homeostasis

Several miRNAs are expressed primarily in the adult human kidney (such as miR-215, miR-146a and miR-886); other miRNAs (for example, miR-192, miR-194, miR-21, miR-200a, miR-204 and let-7a–g), are increased in the kidney as well as in other organs [15]. This expression is tissue-specific and can be dependent on the developmental stage. Deletion of the miR-17~92 cluster leads to defective embryogenesis, affecting progenitor cells and the development of nephrons. Mice deficient in miR-17~92 are categorized by renal hypodysplasia and cause glomerular damage and proteinuria [16].

miRNAs are involved in different renal cellular processes, glomerular haemodynamia, as well as the maintenance of fluid and electrolyte balance. Other miRNAs in the kidney can also affect renin-expressing juxtaglomerular cells, which leads to damage to these cells. Consequently, plasma renin level decreased, and it was associated with hypotension and renal fibrosis [17]. The miRNAs also can help in the osmolarity homeostasis and have effects on the process of Na^+^, K^+^ and Ca^2+^ regulation in the condition of hypertonic environments [18].

## 3. Role miRNA in DKD: Murine Experiments

Hyperglycaemia can trigger a complex interplay between metabolic and hemodynamic factors leading to the genesis of various diabetic complications, including DKD. The presence of high glucose concentration has an adverse influence on all renal cell lineages (mesangial cells, tubular cells, podocytes and endothelial cells) and can modulate the expression of miRNAs affecting cellular intercommunication and kidney tissue homeostasis [19].

Numerous studies have demonstrated the difference in the expression of circulating miRNAs throughout the progression of DKD [15]. A high glucose condition affects the expression of miR-29a and miR-29c, leading to the apoptosis of podocytes and the promotion of pro-fibrotic substances [20,21,22]. The hyperglycemia also regulates the expression of miR-25, miR-93 and miR-192, which in turn affects the redox system, vascular endothelial growth factor and tubulointerstitial fibrosis [23,24,25]. A comprehensive description of changes in the expression of 41 miRNAs in different animal studies is summarized in Table 1.

## 4. Urinary miRNA Research: Human Evidence

Research into miRNA has unveiled obscure puzzles of the pathophysiology of DKD. Furthermore, other investigators have interrogated the clinical utility of these miRNAs for routine practice. Emerging evidence indicates the probable roles of urinary miRNAs as predictors of DKD development or progression [66]. Urine miRNA levels provide a direct reflection of kidney tissue damage. These miRNAs originate from cells and are encapsulated into extracellular vesicles, named exosomes, and are secreted in various biological fluids, including urine. Exosomes can exert paracrine effects and serve as a mediator of intercellular communication. They are stable in biological fluid as well as in paraffin-embedded sections, rendering these exosomes suitable as “liquid biopsy” or biomarkers of specific disease conditions [67]. A number of studies have examined the relationship between urinary microRNA and blood sugar levels. They found that concentrations of various urinary extravesical miRNAs (such as miRNA-941-5p, miRNA 34c-5p and miRNA-208a-3p) were correlated with levels of glycosylated hemoglobin [68]. Table 2 lists changes in the expression of urinary miRNA associated with DKD in clinical research. We identified 141 unique urinary miRNAs associated with DKD. Peculiarly, the direction of expression of specific miRNA differs between types of DM (type 1 vs. 2) and also between distinct patient cohorts (as highlighted in bold). A possible explanation might reside in unclear mechanisms between the two types of DM. In addition, several technical concerns may affect the miRNA profiling, including methods of specimen collection (processing and storage), a fraction of extracted urine (urine, urinary extra-vesicle, extra-vesicle depleted urine fraction), analytic platform (qPCR, microarray, genome-wide profiling by small-RNA sequencing) and artifact contamination [68]. Finally, the cell source of urinary miRNA can arise from any genitourinary tract, and the function of urinary miRNA is not necessarily the same as circulating ones. Sophisticated bioinformatics analysis and network interaction maps are capable of identifying Gene Ontology processes, classification and relevant pathways of target genes in this modern era [66,69]. These approaches may help to decipher the biological functions of urinary miRNA in the pathophysiology of DKD.

## 5. Performances of Urinary miRNAs as Disease Biomarker

A body of literature attempted to identify potential biomarkers from urine specimens in predicting the degree of kidney damage. Individual miRNAs or a cluster of miRNAs were used as possible biomarkers of DKD with satisfactory prediction performance. Li et al. found that urinary expression of let-7c-5p, miR29c-5p and miR-15b-5p could predict DKD with the area under curves (AUC) of 0.818, 0.774, and 0.818, respectively [70]. Eissa S et al. observed that the AUC of miR-15b, miR-34a, and miR-636 were 0.883, 0.917 and 0.984, respectively, for distinguishing DKD from controls. The panel composed of three miRNAs yielded an AUC of 0.912 [78,81]. The expressed levels of miR-95-3p, miR-185-5p, miR-1246, and miR-631 in urinary sediments can also yield a good accuracy (AUC, 0.863) for differentiating DKD from non-DKD or other disease conditions [88]. Collectively, all this evidence suggests the potential use of urinary miRNAs as non-invasive biomarkers for predicting DKD.

## 6. Conclusions

Sensitive biomarkers to guide decision-making in the management of DKD remain urgently awaited. Urinary miRNA may represent promising non-invasive, and cheap means with diagnostic or predictive implications in DKD. However, the clinical application is unsatisfactory to date because of inconsistencies between reported data. Most of the research projects reported were discovery experiments with small samples and limitation validation. In addition, the discrepancies may be in part explained by differences in procedures of urine isolation, the proportion of urine fraction (fresh urine, concentrates of extra-vesicles or extra-vesicle depleted fraction) used, heterogeneity in reporting outcome and in the degree of kidney severity (micro vs. macroalbuminuria, intermittent vs. persistent proteinuria). Furthermore, the advances in analytic methodology enabled the discovery of new miRNAs. Recently, the introduction of the sensor-based methodology using labels on magnetic beads can magnify measurement power. The incorporation of the use of spectrophotometry or electrochemistry, rather than direct visualization, can further enhance the quantification accuracy [89] with small sample sizes and limited validation. Consensus and standardization of methodologies applied to retrieve, isolate, store and measure miRNAs may enhance the reproducibility of the study results. Further validation with an extended sample size is warranted to move the field forward clinical translation of urinary miRNA in DKD.

## 7. Review Criteria

We conducted a narrative review of animal and human studies in published literature from 1 October 2013 until 30 July 2022. The PubMed database was used for searching the scientific literature using the following search terms: “microRNA”, “diabetic kidney disease”, “diabetic nephropathy”, and “urinary”. This narrative literature review primarily focused on original articles written in the English language and published in peer-reviewed journals. The reference lists of included articles were also hand-searched. References were managed using EndNote 20.1.

Independent researchers (CKH and YTC) managed titles and abstracts to identify potentially eligible studies for full-text review. We only included original articles and case series with over two cases in the English language for further assessment. Data extraction was performed in duplicate by two independent reviewers (CCL, KJY). When multiple articles reporting data from the same study population were identified, the most comprehensive data were used. All reported miRNAs are indexed and annotated as a mature sequence of miRNA identified from the miRbase database. Studies were required to provide quantizable data on the expression levels of miRNA, such as fold changes (down-expression, up-expression, not change or absolute value) or area under curves. We contacted the study authors regarding possible incomplete data on miRNA quantities presented in selected publications. The methodological quality of included studies was not assessed in this narrative review because large heterogenicity presented in the testing techniques (ELISA, microarray, q-PCR, sequencing, etc.) and differences in reporting outcomes.

**Figure 1:**
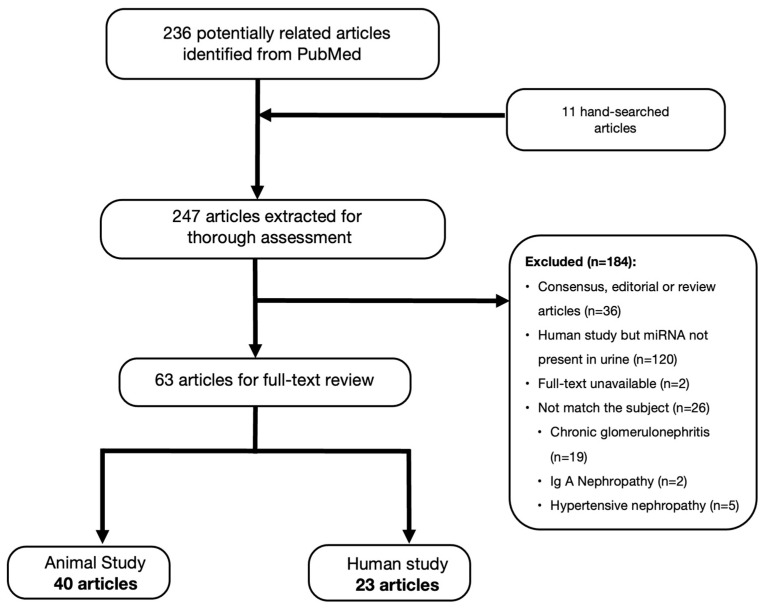
Flow chart of literature search and selection.

We identified 247 records from the PubMed database for the initial assessment, and only 63 articles were included for full-text review; 40 articles related to animal studies and 23 human research articles, respectively (Figure 1). This literature review included a thorough assessment of 41 miRNA from animal experiments and 141 miRNA from urine samples of DKD patients.

## Figures and Tables

**Table 1:** miRNA implicated in the diabetic animal model.

Mature Sequence of miRNA	Species	Experimental Model	Expression	Number of Study	References
hsa-let-7i-5p	Mouse	Db mice	Up	1	[26]
hsa-miR-129-1-3p	Mouse	Db mice	Down	1	[26]
hsa-miR-130a-3p	Mouse, cell	Db mice, HepG2 cells	Up	1	[27]
hsa-miR-133b	Rat	High-Fat Diet/Streptozotocin-Induced Diabetic Rat	Up	1	[28]
hsa-miR-134-5p	Mouse, cell	C57BL/KsJ db mice, Db/Db mice, podocytes	Up	1	[29]
hsa-miR-146a-5p	Mouse, cell	C57BL/6J, miR-146a−/− mice, Podocytes, HK-2 human kidney cells, Db mice, peritoneal macrophages	Up, Down	3	[30,31,32]
hsa-miR-148b-3p	Rat, cell	Proximal tubular cells	Down	1	[33]
hsa-miR-16-5p	Rat	Male Wistar rats	Up	1	[34]
hsa-miR-181a-5p	Rat, cell	HK-2 cells, Otsuka-Long-Evans-Tokushima-Fatty rats	Down	1	[35]
hsa-miR-192-5p	Mouse	Db mice, Ets-1–deficient mice	Up	1	[36]
hsa-miR-200c-3p	Mouse, cell	Db mice, M4200 cells	Down	3	[26,37,38]
hsa-miR-21-5p	Mouse, rat, cell	C57BI/6J mice, miR-21-KO mice, DBA/2J mice, Db mice, kk-ay mice, epithelial-to-mesenchymal, podocytes	Up, Down	7	[39,40,41,42,43,44,45]
hsa-miR-217-5p	Cell	Glomerular mesangial cells	Up	1	[46]
hsa-miR-218-5p	Mouse, cell	Db mice, podocytes	Up, Down	2	[26,47]
hsa-miR-23c	Rat, cell	HK-2 cells	Down	1	[48]
hsa-miR-26a-5p	Rat, cell	OLETF rats, NRK-52E cells, glomerular, mesangial cells, podocytes, proximal tubular epithelial cells	Up, Down	2	[49,50]
hsa-miR-29a-3p	Mouse, cell	Db mice, Smad3-knockout (KO) db mice, mouse embryonic fibroblasts, NRK52E cells, mesangial cells	Down	2	[51,52]
hsa-miR-29a-5p	Mouse	Male FVB mice	Down	1	[53]
hsa-miR-29c-3p	Mouse	Db mice	Down	1	[26]
hsa-miR-30a-5p	Rat	High-Fat Diet/Streptozotocin-Induced Diabetic Rat	Up	1	[28]
hsa-miR-335-5p	Mouse	Male C57BL/6J mice	Up	1	[54]
hsa-miR-33a-5p	Mouse, cell	Db mice, MMCs (CRL1927) and HEK293 cells	Down	1	[55]
hsa-miR-342-5p	Rat	High-Fat Diet/Streptozotocin-Induced Diabetic Rat	Up	1	[28]
hsa-miR-34a-5p	Mouse, cell	Db mice, mesangial cells, HK-2cells, podocytes	Down	3	[56,57,58]
hsa-miR-375-5p	Mouse	Male C57BL/6J mice	Up	1	[54]
hsa-miR-378d	Mouse	Db mice	Up	1	[26]
hsa-miR-451a	Mouse, rat	Db mice, male Wistar rats	Down	2	[34,59]
hsa-miR-7977	Mouse	Db mice	Down	1	[26]
hsa-miR-92b-5p	Rat	Lean Zucker, obese Zucker rats	Down	1	[60]
hsa-miR-93-5p	Mouse, cell	Pod-iCreERT2 mice, podocytes	Down	1	[61]
hsa-miR-99b-5p	Mouse, rat, cell	Db mice, mesangial cells, proximal tubular epithelial cells	Down	4	[62,63,64,65]

miRNAs are annotated as a mature sequence of miRNA identified from the miRbase database.

**Table 2:** Urinary miRNA implicated in human studies related to diabetic kidney disease.

Mature Sequence of miRNA	Sample	DM Type	Patient Number	Urinary Expression in DKD	References
has-let-7a-3p	Blood/Urine	2	27	Down	[66]
hsa-let-7c-5p	Urine	2	63	Up	[70]
hsa-let-7f-1-3p	Blood/Urine	2	27	Down	[66]
hsa-let-7i-5p	Urine	2	160	Up	[71]
hsa-miR-106b-3p	Blood/Urine	2	27	Down	[66]
hsa-miR-10a-5p	Urine	1	48	Up vs. PMA	[68]
hsa-miR-10b-5p	Urine	1	48	Up vs. PMA	[68]
hsa-miR-1224-3p	Urine	1	40	Up	[72]
hsa-miR-122-5p	Urine	1	48	Up	[68]
hsa-miR-126	Urine	2	92	Up	[73]
hsa-miR-126-5p	Urine/blood	1	147	Down	[74]
hsa-miR-1275	Urine	2	6	Up	[75]
hsa-miR-1307-3p	Urine	1	48	Down	[68]
hsa-miR-130a-3p	Urine	1	48	Up	[68]
hsa-miR-130a-5p	Tissue/Urine	1	24	Up	[76]
hsa-miR-133a-3p	Urine	1	48	Up	[68]
hsa-miR-133a-3p	Urine	2	6	Down	[77]
hsa-miR-133b	Urine	2	220	Up	[78]
hsa-miR-135b-5p	Urine	2	160	Up	[71]
hsa-miR-141-3p	Urine	1	40 + 48	Up	[68,72]
hsa-miR-142-3p	Urine	1	48	Up	[68]
hsa-miR-145-5p	Tissue/Urine	1	24	Up	[76]
hsa-miR-146	Urine	2	92	Up	[73]
hsa-miR-148a-3p	Urine	1	48	Up	[68]
hsa-miR-148b-3p	Urine	2	56	Down	[79]
hsa-miR-150-3p	Urine	2	6	Up	[77]
hsa-miR-150-5p	Urine	2	80	Up	[80]
hsa-miR-152-3p	Urine	1	48	Up	[68]
hsa-miR-153-3	Urine	2	6	Down	[77]
hsa-miR-155	Urine	2	92	Up	[73]
hsa-miR-155-5p	Tissue/Urine	1	24	Down	[76]
hsa-miR-1587	Urine	2	6	Up	[75]
hsa-miR-15a-5p	Urine	2	80	Down	[80]
hsa-miR-15b-5p	Urine	2	232 + 63 + 160	Up/Down/Down	[70,71,81]
**hsa-miR-17-5p**	**Urine**	**2**	**56**	**Down**	**[79]**
**hsa-miR-17-5p**	**Urine**	**1**	**40**	**Up**	**[72]**
hsa-miR-181a-5p	Urine	1	48	Up	[68]
hsa-miR-183-5p	Urine	1	48	Up	[68]
hsa-miR-188-3p	Urine	1	40	Down	[72]
**hsa-miR-188-5p**	**Urine**	**1**	**48**	**Down**	**[68]**
**hsa-miR-188-5p**	**Urine**	**2**	**6**	**Up **	**[77]**
hsa-miR-190a-5p	Blood/Urine	2	27	Down	[66]
hsa-miR-1912	Urine	1	40	Up	[72]
hsa-miR-1913	Urine	1	40	UP	[72]
**hsa-miR-192-5p**	**Urine**	**2**	**56**	**Down**	**[79]**
**hsa-miR-192-5p**	**Urine**	**1**	**48**	**Up**	**[68]**
hsa-miR-193b-5p	Urine	2	6	Down	[69]
hsa-miR-196a	Urine	2	209	UP	[82]
**hsa-miR-197-3p**	**Urine**	**2**	**160**	**Down**	**[71]**
**hsa-miR-197-3p**	**Urine**	**1**	**48**	**Down**	**[68]**
hsa-miR-200a-3p	Urine	1	48	Up	[68]
hsa-miR-200c-3p	Urine	1	48	Up	[68]
hsa-miR-2117	Urine	2	6	Up	[75]
hsa-miR-214-3p	Urine	1	40	UP	[72]
**hsa-miR-21-5p**	**Urine**	**2**	**56**	**Down**	**[79]**
**hsa-miR-21-5p**	**Urine/blood**	**1**	**147**	**Up**	**[74]**
**hsa-miR-216a-5p**	**Urine**	**2**	**56**	**Down**	**[79]**
**hsa-miR-216a-5p**	**Urine**	**1**	**50**	**Down**	**[83]**
hsa-miR-217-5p	Urine	2	56	Down	[79]
hsa-miR-219a-3p	Urine	2	6	Up	[75]
hsa-miR-221-3p	Urine	1	40	Down	[72]
hsa-miR-222-3p	Urine	1	48 + 40	Up	[68,72]
hsa-miR-22-3p	Urine	1	48	Up	[68]
hsa-miR-23c	Urine	2	6	Up	[77]
hsa-miR-24-3p	Urine	2	160	Up	[71]
hsa-miR-27b-3p	Urine	2	160	Down	[71]
hsa-miR-29a-3p	Urine	2	83	NC	[84]
hsa-miR-29a-5p	Urine	2	83	Up	[84]
hsa-miR-29b-1-5p	Urine	1	40	Up	[72]
**hsa-miR-29c-3p**	**Urine**	**2**	**220 + 56 + 63**	**Up/Up/Down**	**[70,78,79]**
**hsa-miR-29c-3p**	**Urine**	**1**	**48**	**Up**	**[68]**
**hsa-miR-29c-5p**	**Urine**	**2**	**83**	**NC**	**[84]**
**hsa-miR-29c-5p**	**Tissue/Blood/Urine**	**2**	**27 + 16**	**Down**	**[66,85]**
hsa-miR-30a-3p	**Urine**	**2**	**160**	**Up**	**[71]**
**hsa-miR-30a-5p**	**Urine**	**1**	**48 + 27**	**Up/Down**	**[68,86]**
hsa-miR-30b-5p	Blood/Urine	2	27	Down	[66]
hsa-miR-30c-5p	Blood/Urine	**2**	**27**	**Down **	**[66]**
hsa-miR-30d-5p	Blood/Urine	2	27	Down	[66]
hsa-miR-30e-3p	Blood/Urine	2	27	Down	[66]
hsa-miR-3137	Urine	2	6	Down	[69]
hsa-miR-31-5p	Urine	1	48	Up	[68]
hsa-miR-3168	Urine	1	48	Down	[68]
hsa-miR-3184-3p	Urine	1	48	Up	[68]
hsa-miR-320b	Blood/Urine	2	27	Up	[66]
hsa-miR-320c	Urine	2	41	Up	[87]
hsa-miR-320e	Urine	2	6	Up	[77]
hsa-miR-323b-5p	Urine	1	40	Down	[72]
hsa-miR-331-3p	Blood/Urine	2	27	Down	[66]
hsa-miR-335-5p	Urine	1	40	Up	[72]
hsa-miR-339-3p	Urine	1	48	Up	[68]
hsa-miR-342-3p	Urine	1	48	Up	[68]
hsa-miR-342-5p	Urine	2	220	Up	[78]
hsa-miR-34a-5p	Urine	2	232	Up	[81]
hsa-miR-362-3p	Urine	2	80	Up	[80]
hsa-miR-362-5p	Urine	1	48	Down	[68]
hsa-miR-363-3p	Urine	1	27	Down	[86]
hsa-miR-3677-3p	Urine	2	6	Up	[77]
hsa-miR-373-5p	Urine	1	40	Down	[72]
hsa-miR-375-5p	Urine	2	160	Down	[71]
**hsa-miR-377-5p**	**Urine**	**1**	**50**	**Up**	**[83]**
**hsa-miR-377-5p**	**Urine**	**2**	**56**	**Up**	**[79]**
hsa-miR-424-3p	Urine	1	48	Down	[68]
**hsa-miR-424-5p**	**Tissue/Urine**	**1**	**40 + 24 + 27**	**Up/Down/Down**	**[72,76,86]**
hsa-miR-4270	Urine	2	6	Up	[69]
hsa-miR-4286	Urine	1	48	Down	[68]
hsa-miR-429	Urine	1	40	UP	[72]
hsa-miR-433	Urine	1	40	Up	[72]
hsa-miR-4491	Urine	2	6	Up	[75]
hsa-miR-4507	Urine	2	6	Up	[75]
hsa-miR-4516	Urine	2	6	Up	[75]
hsa-mir-453	Urine	1	40	Down (Up in persistent microalbuminuria)	[72]
hsa-miR-4534	Urine	2	6	Up	[75]
hsa-miR-4687-3p	Urine	2	6	Up	[75]
hsa-miR-486-3p	Urine	1	40	Up	[72]
hsa-miR-486-5p	Urine	1	27	Up	[86]
hsa-miR-495-5p	Urine	1	27	Down	[86]
hsa-miR-498	Urine	2	6	Up	[75]
hsa-miR-5007-3p	Urine	2	6	Up	[75]
hsa-miR-500a-5p	Urine	2	160	Down	[71]
hsa-miR-5088-5p	Urine	2	6	Up	[75]
hsa-miR-5091	Urine	2	6	Up	[75]
hsa-miR-516b-5p,	Urine	2	6	Up	[75]
hsa-miR-520h	Urine	1	40	Down	[72]
hsa-miR-524-5p	Urine	1	40 + 27	Down/Down	[72,86]
hsa-miR-548ah-3p	Urine	2	6	Up	[77]
hsa-miR-548p	Urine	2	6	Up	[77]
hsa-miR-552-5p	Urine	1	40	Up	[72]
hsa-miR-589-5p	Urine	1	40	Down	[72]
hsa-miR-6068	Urine	2	41	Up	[87]
hsa-miR-6076	Urine	2	41	NC	[87]
hsa-miR-616-5p	Urine	1	27	Up	[86]
hsa-miR-619-5p	Urine	1	40	Up	[72]
hsa-miR-628-5p	Urine	1	40	Up	[72]
hsa-miR-636	Urine	2	232	Up	[81]
hsa-miR-638	Urine	1	40	UP	[72]
hsa-miR-640	Urine	1	27	Up	[86]
hsa-miR-645	Urine	1	27	Up	[86]
hsa-miR-665	Urine	1	27	Down	[86]
hsa-miR-6809-5p	Urine	2	6	Down	[69]
hsa-miR-6831-5p	Urine	2	6	Up	[69]
hsa-miR-760	Urine	2	6	Up	[77]
hsa-miR-765	Urine	1	40	UP	[72]
hsa-miR-767-3p	Urine	1	27	Down	[86]
hsa-miR-770-5p	Urine	1	27	Up	[86]
hsa-miR-7846-3p	Urine	2	6	Up	[69]
hsa-miR-877-3p	Urine	2	80	Up	[80]
hsa-miR-92a-3p	Urine	1	48 + 40	Up/Down	[68,72]
hsa-miR-92b-5p	Urine	1	40	UP	[72]
hsa-miR-93-5p	Urine	2	160	Up	[71]
hsa-miR-98-3p	Blood/Urine	2	27	Down	[66]
**hsa-miR-99b-5p**	**Urine**	**1**	**48**	**Up**	**[68]**
**hsa-miR-99b-5p **	**Blood/Urine**	**2**	**27**	**Down **	**[66]**

miRNAs are annotated as a mature sequence of miRNA identified from the miRbase database. NC: not changed. miRNA in bold indicates differentially expressed direction between publications.

## Data Availability

Not applicable.
